# Delayed Hemolytic Transfusion Reaction With Acute Kidney Injury Due to Anti-E Antibody in a 43-Year-Old Woman With Iron-Deficiency Anemia

**DOI:** 10.7759/cureus.93634

**Published:** 2025-10-01

**Authors:** Kushal Panja, Rajeev Upreti

**Affiliations:** 1 General Medicine, North Manchester General Hospital, Manchester University National Health Service (NHS) Foundation Trust, Manchester, GBR

**Keywords:** alloimmunization, anti-e antibody, delayed hemolytic transfusion reaction, extravascular hemolysis, packed red blood cell transfusion

## Abstract

Delayed hemolytic transfusion reactions (DHTRs) are uncommon yet serious complications of blood transfusion and are often underdiagnosed in patients with negative pre-transfusion antibody screens and a negative direct antiglobulin test (DAT). We report a case of a 43-year-old woman with iron-deficiency anemia secondary to menorrhagia who developed a DHTR with acute kidney injury following red blood cell transfusion. Despite a negative pre-transfusion antibody screen and DAT, she developed hemolysis and renal dysfunction approximately one week post-transfusion; the reference laboratory later identified anti-E antibodies. This case highlights the diagnostic challenges of DHTRs when pre-transfusion serology and DAT are negative. Vigilant clinical suspicion and post-transfusion monitoring are crucial to prevent and manage potentially life-threatening complications.

## Introduction

Delayed hemolytic transfusion reactions (DHTRs) typically occur more than 24 hours after red blood cell (RBC) transfusion, most often within five to 14 days, but occasionally up to 30 days, and are mediated by an anamnestic immune response to a previously encountered RBC alloantigen [1-3]. Pathophysiologically, alloantibodies bind to transfused RBC antigens, leading to their destruction either through the reticuloendothelial system (extravascular hemolysis) or, in the case of certain complement-fixing antibodies, by direct lysis in the circulation (intravascular hemolysis) [4]. ABO antibodies, typically naturally occurring IgM, are most often implicated in acute hemolytic transfusion reactions. In contrast, Rh antibodies, particularly anti-E and anti-c, are among the most frequent causes of DHTR [5].

The direct antiglobulin test (DAT) is a cornerstone in the evaluation of suspected transfusion reactions, as it detects IgG or complement bound to the patient’s RBCs. However, false-negative DAT results are well recognized, occurring when antibody coating is of low density or only transiently detectable, and may lead to diagnostic delay [4].

The reported prevalence of DHTR is approximately one in 2,000 to one in 5,000 transfusions, with a higher risk among chronically transfused patients, such as those with sickle cell disease or thalassemia [6,7]. Nevertheless, DHTR may also occur in otherwise healthy individuals, making clinical vigilance essential [8].

We present a case of anti-E-mediated DHTR in a 43-year-old woman with iron-deficiency anemia, complicated by acute kidney injury (AKI). This case is distinctive for its DAT-negative presentation and renal involvement, both of which remain rarely reported in the literature.

## Case presentation

A 43-year-old female was admitted with fatigue, lightheadedness, and shortness of breath on exertion. These symptoms had temporarily improved following an iron infusion and a two-unit RBC transfusion for symptomatic iron-deficiency anemia at the gynecology department one week prior to this admission (hemoglobin increased from 48 to 72 g/L). She subsequently developed frank hematuria accompanied by persistent fatigue, dizziness, and exertional breathlessness.

The patient had iron-deficiency anemia secondary to ongoing menorrhagia due to uterine fibroids. She had previously undergone a myomectomy for fibroid management. Her regular medications included tranexamic acid, mefenamic acid, and oral iron supplements. She had no known drug allergies. Notably, she had experienced two prior transfusion reactions, although details regarding these episodes and any associated antibody investigations were unavailable.

Upon admission (with stable vitals), baseline laboratory investigations are summarized in Table 1.

**Table 1:** Laboratory investigations +++ strong/marked, ++++ = very strong/maximum positive finding eGFR: estimated glomerular filtration rate, LDH: lactate dehydrogenase, ALT: alanine aminotransferase, ALP: alkaline phosphatase, DAT: direct antiglobulin test

Parameters	Patient values	Reference range
Hemoglobin	65 g/L	115.0-165.0 g/L
Creatinine	95 µmol/L	45-84 µmol/L
eGFR	63 mL/min/1.73 m²	>90 mL/min/1.73 m²
LDH	1280 U/L	139-249 U/L
Reticulocytes	118 × 10⁹/L	20-80 × 10⁹/L
Haptoglobin	<0.10 g/L	0.30-2.00 g/L
ALT	66 U/L	<35 U/L
ALP	85 U/L	30-130 U/L
Total bilirubin	40 µmol/L	<21 µmol/L
DAT	Negative	Negative
Urine (dipstick)	Blood +++, protein ++++	Negative

She reported excessive dizziness, lethargy, thirst, and nocturia. There were no signs of infection, rash, or neurologic deficits. Hematology was consulted; despite a negative DAT and a negative pre-transfusion antibody screen, hemolysis was suspected. Blood samples were sent to the local Regional Centre for Immunohaematology for further testing.

The Regional Transfusion Centre subsequently identified anti-E. A review of transfusion records showed that one of the units transfused pre-admission was E-positive, consistent with an anamnestic anti-E response and DHTR. Renal function declined transiently (compatible with hemoglobinuria-associated AKI) but stabilized with supportive care. Notably, the DAT initially remained negative, a significant diagnostic pitfall reported in DHTR.

She was managed conservatively with frequent monitoring of hemoglobin and renal function. Within a week, the hemoglobin level improved to 81 g/L, and kidney function normalized. She was discharged with hematology follow-up; iron deficiency was treated with further IV iron infusions. For any future transfusion needs, extended antigen matching and provision of E-negative RBCs were recommended.

## Discussion

DHTRs occur when a previously sensitized patient is re-exposed to the relevant red cell antigen, triggering an anamnestic antibody response and hemolysis several days to weeks after transfusion [1-3]. In most cases, DHTRs are characterized by extravascular hemolysis, where antibody-coated RBCs are opsonized and phagocytosed by splenic or hepatic macrophages via Fc receptor or complement receptor pathways [4]. However, intravascular hemolysis can occur when complement-fixing antibodies (such as those directed against Rh, Kidd, or certain Kell antigens) activate the classical complement cascade, leading to MAC-mediated red cell lysis directly in the circulation [9,10]. This process results in the release of free plasma hemoglobin, which contributes to the scavenging of nitric oxide, oxidative stress, and endothelial dysfunction [10]. In our patient, the findings of hemoglobinuria, markedly elevated lactate dehydrogenase, and depressed haptoglobin strongly support a predominant intravascular hemolysis component, with a minor extravascular contribution suggested by transient bilirubin elevation.

Anti-E is a common, clinically significant Rh alloantibody frequently identified among alloimmunized patients across diverse populations and has been implicated in DHTR [11,12]. Although a positive DAT is classically expected, DAT-negative DHTR is well recognized and may occur when antibody-coated cells are present at low density or only transiently detectable, which can result in diagnostic delay [3,4]. Beyond the more common antibodies such as Rh and Kidd, rare causes of DHTR have also been described. Case reports document hyperhemolysis syndrome due to anti-M in a patient with myelofibrosis [13] and DHTR in sickle cell disease [14], as well as reactions associated with antibodies to less frequently encountered antigens, including Duffy and Diego systems [15-17]. These reports highlight the clinical heterogeneity of DHTR and the challenges in establishing the exact etiology in practice, particularly when molecular testing is not performed. Against this backdrop, our case is distinctive for its DAT-negative presentation with anti-E alloantibody and the additional complication of AKI, which remains a rarely reported feature.

AKI is an uncommon but recognized complication of hemolytic transfusion reactions, thought to result from hemoglobin-mediated tubular toxicity and pigment nephropathy during hemoglobinuria [1]. Our patient’s transient renal impairment and dark urine are consistent with this mechanism.

The severity of DHTR is best considered along a clinical spectrum. Mild cases may present only with subtle anemia or biochemical evidence of hemolysis. In contrast, severe cases include hyperhemolysis syndrome, in which both transfused and autologous RBCs are destroyed, sometimes precipitating life-threatening anemia [18]. Our patient’s clinical course was moderate, with biochemical hemolysis, symptomatic anemia, and transient AKI, but without the catastrophic features of hyperhemolysis.

Management of DHTR centers on prompt recognition, supportive care, and prevention of further antigen exposure. For patients with a history of alloimmunization or prior reactions, transfusing phenotype-matched, antigen-negative RBC units is recommended to reduce recurrence risk [19,20]. In severe cases (e.g., hyperhemolysis), immunomodulatory therapies such as corticosteroids or IV immunoglobulin, and in selected refractory scenarios, complement inhibition (e.g., eculizumab) may be considered, as per expert reviews [19,20].

## Conclusions

This case illustrates the diagnostic challenges of DHTRs, particularly when initial antibody screening and the DAT are negative. The presence of anti-E alloantibody and the development of AKI highlight the potential severity of these reactions, even in patients without complex comorbidities. Careful attention to clinical presentation, laboratory trends, and transfusion history is crucial for ensuring timely recognition. Collaboration with transfusion medicine services enables the accurate identification of causative antibodies, guiding the use of antigen-negative, phenotype-matched RBC units and thereby reducing the risk of recurrence. Greater awareness of DAT-negative DHTRs may help prevent delays in diagnosis and improve outcomes in future patients.

From a broader clinical perspective, this case reinforces the importance of vigilance when managing patients who require repeated transfusions, particularly those with gynecological, hematological, or chronic medical conditions. Strengthening communication between clinicians and transfusion services, along with early consideration of extended antigen typing, can play a vital role in improving transfusion safety and minimizing the risk of adverse outcomes.
